# Moderations in performance, immunity, tissue architecture, and vaccine viability induced by water magnetization in broiler farms

**DOI:** 10.14202/vetworld.2021.1695-1710

**Published:** 2021-06-30

**Authors:** Essam S. Soliman, Rania T. Hamad, Rania A. Hassan

**Affiliations:** 1 Animal, Poultry, and Environmental Hygiene Division, Department of Animal Hygiene, Zoonosis, and Animal Behavior, Faculty of Veterinary Medicine, Suez Canal University, Ismailia 41522, Egypt; 2 Department of Pathology, Faculty of Veterinary Medicine, Menoufia University, Al Minufya 32511, Egypt; 3 Animal Production Division, Department of Animal Wealth Development, Faculty of Veterinary Medicine, Suez Canal University, Ismailia 41522, Egypt

**Keywords:** broilers, immunity, magnetic water, microbial survival, Newcastle vaccine viability, water impurities

## Abstract

**Background and Aim:**

Water magnetization contributes to increased molecular ionization and fluidity, which improves biological activities. This study tests the influence of magnetic water on the viability of the Newcastle vaccine and the survival of *Escherichia coli* and *Salmonella*Typhimurium, as well as the influence of magnetic water in face of water impurities’ challenges on performance, immunity, and tissue architecture in broiler chickens.

**Materials and Methods:**

An *in vitro* 96-micro-well plate minimal inhibitory concentration was utilized to test the influence of water, saline, and magnetic water on Newcastle vaccine viability and *E. coli* O157:H7 and *S*. Typhimurium survival. The 245 experimental 1-day-old female Ross^®^ 308 broilers used in this study were divided into seven groups of 35 birds each. Broilers were provided with magnetic drinking water (13,200 gausses) for 6 h daily from the 5^th^ day and were challenged on days 14, 21, 28, and 35 using sodium chloride (700 mg/L), calcium sulfate (80 mg/L), lead acetate (500 mg/L), yeast extract 5% (5 mg/L), diazinon (2.5 mL/L), and *E. coli O157:H7* (1.6 × 10^9^ CFU/mL). A total of 2040 samples (96 diluent-Newcastle virus vaccine mixes, 96 microbial-magnetic water mixes, 231 sera, 231 intestinal swabs, and 1386 organ samples) were collected.

**Results:**

An *in vitro* trial revealed highly significant (p<0.01) declines of 94.13%, 84.53%, and 10.31% in the Newcastle vaccine titer in water, saline, and magnetic water, respectively, and 54.91% and 39.89% in *E. coli* O157:H7 and *S*. Typhimurium survival, respectively, after 4 h. In all challenged groups, broilers exhibited highly significant (p<0.01) increases in performance, carcass/organs weight, immunoglobulin G, immunoglobulin M, and *Lactobacillus* counts; significant improvement in tissue architecture and biochemical parameters; and highly significant (p<0.01) reductions in cortisol, superoxide dismutase, and total bacterial and Enterobacteriaceae counts.

**Conclusion:**

Magnetic water could maintain vaccine viability and vaccination efficiency, reduce microbial survival, and minimize the negative influence of all induced challenges.

## Introduction

Water is an essential element for the livability, health, and performance of broilers. Many physiological functions such as food digestion, metabolism, regulation of body temperature, and excretion of waste and toxic substances involve water in their normal biological process. Broilers normally consume water at a rate of 1.5:2 times as much as they consume feed [1]. Providing *ad libitum* access to water of adequate quality [2], taste [3], and temperature can maintain the flock’s performance [4]. As a best practice procedure in poultry drinking water sanitation management, water chlorination is carried out in broiler farms to minimize bacterial contamination and biofilm formation, reduces infectious agent spread, and provides water of high quality [5]. Water has potential hydrogen-bonding capacity, acts as a solvent for many organic and inorganic substances, and is an ideal media for the growth and multiplication of pathogenic microorganisms. Impurities such as herbicides, pesticides, organic and inorganic chemicals, fertilizers, and microorganisms influence drinking water quality on broiler farms [6], which gain access to water resources from the ground and process it for use as poultry drinking water. Many epidemiological surveys have revealed the isolation of different pathogenic microorganisms from drinking water provisions for birds on broiler farms, including *Escherichia coli*, *Vibrio*, *Salmonella*, *Shigella, Klebsiella, Proteus, Pseudomonas, Staphylococci, Streptococci, Aspergillus, Mucor*, and *Penicillium* [7-9]. Once these impurities and contaminants enter the broiler’s drinking water, they exert a detrimental influence on the bird’s performance and immunity [10].

In recent years, magnetic treatment of water has become widely used on poultry farms to achieve various targets, including the enhancement of poultry production and health [11,12]. Changes in the biological properties of natural water after magnetization could improve water quality by increasing electric conductivity and the dielectric constant [13]. Physics has shown that water changes weight under the influence of the magnetic field, as more hydroxyl (OH-) ions are created to form alkaline molecules that reduce acidity [11]. Consumption of alkaline water has several beneficial effects on poultry health [14]. However, a permanent magnet could manipulate the structure of water to activate and ionize water molecules and achieve magnetization. Magnetic water can support the formation of a beneficial structure, increase fluidity [15], and improve biological activities [16]. Magnetic water could increase protein and lipid metabolism, alter blood glucose concentrations through manipulating glycolysis and glycogenesis [17], and increase poultry productivity and survival [18]. Magnetization also alters many biological characteristics of water, thus reflecting beneficial effects on poultry, such as shortening the fattening period of broilers, increasing growth rate, improving meat quality [19], minimizing mortalities and diseases [20], and decreasing food consumption while increasing food conversion ratios [21].

The current approach achieves the best results for a study of the *in vitro* influence of magnetic water (13,200 gausses) on Newcastle vaccine viability, and *E. coli* O157:H7 and *Salmonella* Typhimurium survival. This study also aimed to evaluate the *in vivo* effectiveness of magnetic water in neutralizing water impurities. The tested impurities were categorized as inorganic elements (sodium chloride at 700 mg/L), hardness salt (calcium sulfate at 80 mg/L), heavy metal (lead acetate at 500 mg/L), organic matter (yeast extract 5% at 5 mg/L), organophosphate (diazinon at 2.5 mL/L), and microbial agent (*E. coli O157:H7* at 1.6 × 10^9^ CFU/mL). The study also evaluated the *in vivo* influence of magnetic water on productive performance, certain carcass quality characteristics, biochemical parameters, antioxidant activity, serum cortisol concentrations, immunoglobulin concentrations, intestinal and breast muscle bacterial load, and histopathological architecture (liver, heart, spleen, bursa of Fabricius, intestine, and breast muscle) in Ross^®^ 308 broiler chickens.

## Materials and Methods

### Ethical approval

The Scientific Research Ethics Committee (SREC) in the Faculty of Veterinary Medicine- Suez Canal University-Ismailia - Egypt approved the materials and protocols used in the current study under approval number (2020091).

### Study period and location

The *in vitro* study was carried out from January 1^st^ to the end of February 2020 in Animal, Poultry, and Environmental Hygiene laboratories – Faculty of Veterinary Medicine – Suez Canal University – Ismailia. The *in vivo* study was conducted from March 3, 2020, to April 9, 2020, in one of the Poultry Experimental Units, Faculty of Veterinary Medicine, Suez Canal University, Ismailia.

Performance indices, carcass characteristics, and bacteriological examination were conducted in Animal, Poultry, and Environmental Hygiene laboratories. Antioxidant, hormonal, and immunity assays were conducted in the Clinical Pathology laboratories – Suez Canal University Hospital. Histopathological examination and photo micro graphing of organs and muscles were conducted in the Pathology Department laboratories, Al Minufya.

### *In vitro* evaluation of magnetic water effectiveness

#### Preparation of magnetic water

Water magnetization was conducted using N42 Neodymium Disc Magnets, which were purchased from Future Electronics, Cairo. Each disc magnet was 10 mm in diameter, 1.8 mm thickness, >144 LBS bull force, and 12.9-13.2 remanence, 10.8-12.0 coercivity and was composed of neodymium, iron, and boron (NdFeB); an epoxy-nickel-cobber-nickel coating for effective protection of the magnet against corrosion and acidic conditions; and axial magnetization characteristics. The strength of the magnet was tested using a Tenmars TM-197 AC/DC Magnetic Field Meter Gaussmeter Teslameter Data Logging (0.0~30,000 G). The magnetic field strength was estimated using axial magnetic field sensors (TM-192D Digital Mini Triple Axis 2000Hz EMF ELF) and was found to be 13,200 gausses.

#### Preparation of Newcastle virus vaccine and bacterial cultures

Newcastle virus vaccine (live lentogenic Newcastle virus disease (ND) virus of LaSota 10^6^, MEVAC NDV3-LaSota) was purchased from a veterinary clinic and was reconstituted in 100 mL distilled water. *E. coli* O157:H7 suspension (1.5 × 10^5^ CFU.mL^−1^) and an *S*. Typhimurium lyophilized vial (3.4 × 10^2^ CFU) were purchased from Animal Health Research Institute, Dokki, Cairo. *E. coli* O157:H7 and *S*. Typhimurium were propagated as recommended by Fritz *et al*. [22] through the serial passage using MacConkey broth (Thermo Scientific™ Oxoid™ MacConkey Broth, CM0505, 500 g) and tetrathionate broth (Thermo Scientific™ Oxoid™ Tetrathionate Broth Base, CM0029, 500 g) pre-enrichments and were incubated at 44°C/24 h and 37°C/24 h, respectively. The propagation of *E. coli* O157:H7 and *S*. Typhimurium was achieved by transferring 10 mL from positive pre-enrichment MacConkey and tetrathionate broth tubes, which were dropped onto MacConkey agar (Thermo Scientific™ Oxoid™ MacConkey Agar No.3., CM0115, 500 g) and xylose lysine deoxycholate agar (Thermo Scientific™ Oxoid™ X.L.D. Agar, CM0469, 500 g), respectively, and were incubated at 37°C/24 h. The growing colonies displayed a pink color on MacConkey agar, which is a specific characterization of *E. coli* O157:H7, and red color with a black center on the xylose lysine deoxycholate agar, which is a specific characterization for *S*. Typhimurium. The detected colonies were counted, collected, and resuscitated in tryptone soy broth (Thermo Scientific™ Oxoid™ Tryptone Soya Broth, CM0129, 500 g), providing 1.6 × 10^8^ and 4.8 × 10^4^ CFU.mL^−1^ suspensions, respectively.

#### Evaluation of Newcastle virus vaccine viability in water, saline, and magnetic water

The procedures were carried out using 96-micro-well plates with a minimal inhibitory concentration test, as were recommended by Elshikh *et al*. [23] and Soliman *et al*. [24]. Columns 1:4 were inoculated with 100 μL microclimatic tap drinking water (32 wells), columns 5:8 were inoculated with 100 μL physiological saline (32 wells), and columns 9:12 were inoculated with 100 μL magnetic water (32 wells). The 96 wells were inoculated with 10 μL of a resuscitated live lentogenic ND virus of LaSota 10^6^. At specified intervals of 0.25, 0.5, 1.0, 2.0, and 4.0 h, 10 μL of the mixes were examined for the Newcastle virus vaccine titer using hemagglutination (HA) and HA inhibition tests according to Choi *et al*. [25].

### Testing magnetic water against bacterial cultures

*E. coli* O157:H7 and *S*. Typhimurium survival were tested using a 96-micro-well plate minimal inhibitory concentration technique, as recommended by Elshikh *et al*. [23] and Soliman *et al*. [24]. The procedures were carried out with an initial inoculation of the 96 wells using 100 μL magnetic water. Then, 10 μL of *E. coli* O157:H7 suspension (1.6 × 10^8^ CFU.mL^−1^) was aseptically inoculated for each well of columns 1:6 (48 wells) and 10 μL from *S*. Typhimurium suspension (4.8 × 10^4^ CFU.mL^−1^) to each well of columns 7:12 (48 wells). At specified intervals of 0.25, 0.5, 1.0, 2.0, and 4.0 h, 10 μL of the mixes were transferred into 10 μL tryptone soy broth resuscitation tubes, vortexed, and incubated at 37°C/24 h. Then, 10 μL was transferred aseptically from the resuscitation tubes and dropped onto MacConkey and xylose lysine deoxycholate agar at 37°C/24 h. The typical growing colonies of *E. coli* and *S*. Typhimurium were counted using a darkfield colony counter (R164109 Reichert–Jung Quebec Darkfield 3325 Colony Counter). The survival rates were tested, and the killing percentages were calculated concerning the initial counts.

### *In vivo* evaluation of magnetic water effectiveness

#### Experimental broilers and housing microclimate

A total of 245 1-day-old female Ross^®^ 308 broiler chicks were purchased from Ismailia-Egypt Company. Broilers were received and raised in open housing system units based on a deep litter (Hay), as recommended by Soliman and Hassan [26]. The units’ floor was covered with a thin film of meta-bisulfite 0.5 g/m^2^, as recommended by Soliman *et al*. [27], to enhance broiler performance, minimize the formation, and volatilization of ammonia, reduce moisture percentages, and control microbial survival, growth, and development in the litter. The units were supplied with an artificial continuous lighting regimen for 23 h of light and 1 h of darkness using blue LED lights, according to the method described by Soliman and Hassan [28]. The building was installed with natural ventilation aids, which were placed in V-shaped windows with ceiling fans to encourage normal air convection (stack effect). The units were secured by many biosecurity measures, as recommended by Soliman and Abdallah [29], including a foot dip based on 7.5% phenol, restricted access to units, regulated movement through the units, fly-proof nets, break back traps for rodent control, full coverage of the water resource, dry cleaning followed by wet cleaning using water with a quaternary ammonium compound based cleansing agent, and disinfection using sodium hydroxide 5% followed by a formaldehyde spray applied in 24 h intervals.

Broilers were divided into seven groups of 35 broilers each (five replicates of seven birds). Broilers were brooded on their arrival at 34°C, which was achieved and maintained using halogen 4 tubes, 2400 watt heater and oil, 11 blades, and 2500 watt heater, as described by Soliman *et al*. [30]. Later, the temperature was manipulated and decreased gradually at a rate of 3.5°C per week until an optimum thermoneutral zone of 21-24°C was achieved, that is, by the end of the 3^rd^ week. Broiler chickens were provided with yellow corn and soybean meal-based feed rations to satisfy their basic nutritional requirements, as recommended by National Research Council [31] and according to modifications implemented by Applegate and Angel [32]. The nutritive ingredients of the ration included protein, fat, crude fiber, and metabolized energy comprising approximately 23%, 4.84%, 3.39%, and 3000 kcal/kg, respectively, in the starter ration that was provided for the first fourteen days, and approximately 21%, 5.93%, 3.25%, and 3100 kcal/kg, respectively, in the grower ration, which was provided from the 15^th^ day until the end of the experiment, which was designed to last for 38 days. Broilers were provided *ad libitum* access to de-chlorinated water after the birds were served with magnetic water daily for 6 h in the morning. Avian vaccinations were delivered at the massive mean using drinking water, and they were vaccinated against infectious bronchitis on the 6^th^ day using a live attenuated virus of IB-H120 ≥10^3.5^ (MEVAC IB H120), against infectious bursal disease (Gumboro/IBD) at the 14^th^ and 21^st^ days using a live attenuated virus of VMG91 ≥10^3.0^ (MEVAC IBD 818), and against ND at the 18^th^ and 28^th^ days using a live lentogenic ND virus of LaSota ≥10^6.0^ (MEVAC NDV3-Lasota).

#### Magnetic water supply

Drinking water was magnetized using an N42 Neodymium disc magnet with axial magnetization. The magnetic field strength was 13,200 gausses, as estimated using axial magnetic field sensors (TM-192D Digital Mini Triple Axis 2000Hz EMF ELF). Magnetization was carried out in the early morning, and broilers were provided with *ad libitum* access to magnetic water for 6 h daily starting from the 5^th^ day until the end of the experiment.

#### Water impurity challenges

Broiler groups were challenged as follows: The 1^st^ group (G1) was challenged with sodium chloride at a rate of 700 mg/L; the 2^nd^ group (G2) was challenged with calcium sulfate at a rate of 80 mg/L; the 3^rd^ group (G3) was challenged with lead acetate at a rate of 500 mg/L; the 4^th^ group (G4) was challenged with yeast extract 5% at a rate of 5 mg/L; the 5^th^ group (G5) was challenged with diazinon at a rate of 2.5 mL/L; the 6^th^ group (G6) was challenged with *E coli O157:H7* at a rate of 1.6 × 10^9^ CFU/mL, and the 7^th^ group (G7) was preserved as the control. All challenges were induced on 14^th^, 21^st^, 28^th^, and 35^th^ days.

#### Performance indices

Performance indices were calculated following the methods used by Soliman and Hassan [33]. Weekly feed (FI/g) and water intakes (WI/mL) were calculated for each broiler chick by proportionating the total amount of weekly feed and water consumed in each group for the total number of birds surviving and housed within that group. Weekly live body weights (LBW/g) in each group were estimated by weighing approximately 32 birds per group weekly, and the number of birds was calculated by applying a simple random sampling design according to Thrusfield [34] and Thrusfield and Christley [35], with an expected error of 5%.

n =1.96^2^ P_exp_ (1 - P_exp_)/d^2^

Where n = required sample size, P_exp_ = expected prevalence, d = desired absolute precision. Weekly weight Gains (WG/g) were calculated based on subtracting the final body weights (by the end of the week) from the initial weights (at the beginning of the same week) within each group, feed conversion ratios (FCR%) were proportionating the feed intakes to the weekly WG, and performance index (PI) was proportionating broiler’s body weight per kg to the FCR%.

#### Sampling

A total of 2040 samples (96 diluent-based Newcastle virus vaccine mixes, 96 microbial-magnetic water mixes, 231 sera, 231 intestinal swabs, and 1386 organ and tissue samples, including liver, heart, spleen, bursa of Fabricius, intestinal loops [duodenum], and breast muscles) were collected after sacrificing birds by the end of the experiment. Collected blood samples were held in a water bath (Thermo® water bath Precision series Standard, 20 L, 30 to 100°C, 392 mm, GP 20) at 30°C for 30 min and centrifuged (Fisher^®^Thermo Scientific CL10 Centrifuge w/F-G3 Rotor with a max RPM of 4000) at 4000 rpm for 20 min. None of the hemolyzed sera were harvested in 3-mL-capacity Eppendorf tubes, tested for cortisol hormone and serum glucose concentrations, and stored at −20°C until use in other biochemical, antioxidant, and immunological assays [36]. Sacrificed birds’ carcasses were decapitated and de-feathered. The shank and feet were removed, eviscerated, and weighed, which was expressed as CW/g; edible organs (heart and liver). The immune organs (spleen and bursa of Fabricius) were harvested and weighed, which was expressed as g/kg carcass weight. Organs and tissues such as the heart, liver, spleen, bursa of Fabricius, intestinal loops (duodenum), and breast muscles were preserved in formalin 10% for histopathological examination. Intestinal swabs and breast muscle samples were harvested on 9 mL buffered peptone water (Thermo Scientific™ Oxoid™ Buffered Peptone Water, CM0509B, 500 g) and preserved for bacteriological assessment. Bird carcasses were later hygienically disposed of by burial method using quick lime.

#### Serum analysis for biochemical, antioxidant, and hormonal profile

Collected sera samples (231 sera samples were collected by sacrificing 33 broiler chicks per each group) were examined calorimetrically for the total protein (g/dL), alanine aminotransferase (IU/L), creatinine (mg/dL), glucose (mg/dL), triglyceride (mg/dL), total cholesterol (mg/dL), total antioxidant activity (mM/L), malondialdehyde (nmol/mL), and superoxide dismutase (U/mL) using a Roche COBAS Integra 800 Chemical Analyzer. Meanwhile, a Roche Elecsys 1010 Immunoassay Analyzer was used to measure the serum cortisol hormone (mcg/dL), immunoglobulin G (IgG, mg/dL), and immunoglobulin M (IgM, mg/dL) concentrations.

#### Bacteriological examination

Frozen breast muscle samples (231 muscle samples were collected by sacrificing 33 broiler chicks per each group) were thawed, homogenized in a stomacher (Seward^®^ Lab Blender Stomacher^®^400 Circulator), and preserved in 9 mL buffered peptone water (Thermo Scientific™ Oxoid™ Buffered Peptone Water, CM0509B, 500 g). The bacteriological assay was carried out by subjecting preserved intestinal swabs and breast muscle samples in buffered peptone water to ten-fold serial dilutions up to 10^−8^ to cover all expected levels of microbial growth according to American Public Health Association recommendations [37] and modifications [38]. Swabs and breast muscle samples were examined for total bacterial (TBC), *Lactobacillus*, and Enterobacteriaceae counts.

The total *Lactobacillus* count (TLC) was conducted using a De Man, Rogosa, and Sharpe agar (Thermo Scientific™ Oxoid™ De Man, Rogosa, and Sharpe agar, CM0361, 500 *g*) with carbon dioxide gas-producing kits in an anaerobic jar at 37°C for 24-72 h. The TBC and Enterobacteriaceae counts (TEC) were conducted using a standard plate count agar (SPA, Thermo Scientific™ Oxoid™ Plate Count Agar, CM0325, 500 g) and eosin methylene blue agar (EMB, Thermo Scientific™Oxoid™ Modified Levine Eosine Methylene Blue, CM0069B, 500 g), respectively, at 37°C for 24-48 h. Plates were inoculated using a drop plate technique, as recommended by Kim and Lee [39], and were counted using a darkfield colony counter (R164109 Reichert–Jung Quebec Darkfield 3325 Colony Counter) [40].

#### Histopathological examination

Organs and tissues such as the heart, liver, spleen, bursa of Fabricius, intestinal loops (duodenum), and breast muscles were dissected out for histopathological examination after sacrificing broiler chickens at the end of the experimental procedures. The specimens were then fixed in 10% neutral buffered formalin solution for 24 h, washed in water, dehydrated in a series of ethyl alcohol with different concentrations (70%, 90%, and 100%), cleared in xylene, and embedded and blocked in paraffin wax. The blocks were cut into sections 4-5 mm thick and were stained with hematoxylin and eosin, according to Junqueira and Carneiro [41]. The slides were examined and imaged using a Zeiss AxioPlan microscope (Carl Zeiss Micro-Imaging, Thornwood, NY).

### Statistical Analysis

Statistical analysis was carried out using SPSS version 21 (IBM SPSS Statistics 21, IBM Corp., NY, USA) [42,43]. We statistically analyzed the data and results obtained using one-way analysis of variance to determine the significance in consideration for factors such as different measurements and treatment applications. The statistical model empathized:

Y_ij_=μ + α_j_+ ɛ_ij_

Where Y_ij_ was the measurement of dependent variables; μ was the overall mean; α_j_ was the fixed effect of the treatments, and ɛ_ij_ was the random error. Results showed high significance at (p<0.01), significant at (p≤0.05), and non-significant at (p>0.05). Bacterial counts (TLC, TBC, and TEC) were expressed in logarithmic numbers (Log10 CFU/mL) using Microsoft Excel 2016.

## Results

### *In vitro* Newcastle virus vaccine titer and microbial survival

The Newcastle virus vaccine titer in the *in vitro* trial (Table-1) showed a highly significant (p<0.01) decline as the contact time increased in all diluents used, including de-chlorinated water, physiological saline, and magnetic water. The reduction percentages significantly varied from one diluent to another, as magnetic water exhibited the lowest reduction percentage (10.31%), saline a higher reduction percentage (84.53%), and de-chlorinated water the highest reduction percentage (94.13%) after 4 h contact.

**Table-1:** *In vitro* Newcastle virus vaccine viability (Log_10_ mean±SE) and microbial survival (Log10 mean±SE) in magnetic water at different exposure times.

Contact time /h	ND virus vaccine titer mg.dL^-1^			Microbial survival CFU.mL^-1^	
	
	De-chlorinated water	Physiological saline	Magnetic water	*Escherichia coli* O157: H7	*Salmonella* Typhimurium
0.25	5.996^a^±0.000	5.998^a^±0.000	6.000^a^±0.000	8.266^a^±0.011	4.994^a^±0.001
0.5	3.526^b^±0.001	3.220^b^±0.000	5.838^b^±0.017	6.829^b^±0.008	4.811^b^±0.000
1.0	1.700^c^±0.014	2.194^c^±0.026	5.653^b^±0.016	5.787^c^±0.002	4.478^c^±0.002
2.0	0.700^d^±0.014	1.266^d^±0.031	5.499^c^±0.025	5.001^d^±0.002	3.559^d^±0.004
4.0	0.352^e^±0.014	0.928^e^±0.026	5.381^c^±0.007	4.058^e^±0.002	3.005^e^±0.005
p-value	0.001	0.002	0.001	0.000	0.001

**Calculated reduction percentage from the initial concentration**					

**6.000 (10^6^)**				**9.000 (10^9^)**	**5.000 (10^5^)**
0.25	0.06%	0.03%	0.00%	8.16%	0.12%
0.5	41.23%	46.33%	2.70%	24.11%	3.78%
1.0	71.66%	63.43%	5.78%	35.70%	10.44%
2.0	88.33%	78.90%	8.35%	44.43%	28.82%
4.0	94.13%	84.53%	10.31%	54.91%	39.89%

Means carrying different superscripts in the same column are significantly different at (p≤0.05) or highly significantly different at (p<0.01). Means carrying the same superscripts in the same column are non-significantly different at p<0.05. SE=Standard error

Magnetic water significantly influenced *E. coli* O157:H7 with highly significant (p<0.01) reduction rates of up to 54.91% after 4 h. *S*. Typhimurium revealed highly significant (p<0.01) reductions with a lower reduction rate of 39.89% after 4 h compared to *E. coli* O157:H7.

### Performance indices

Monitoring broiler chickens revealed a low total mortality rate of up to 3.26% (eight out of 245 broiler chickens). Postmortem examination of these mortalities confirmed unspecific deaths with common signs such as hyperemia in the intestinal loops, congested liver, and hypertrophied spleen.

LBW in Table-2 shows highly significant (p<0.01) increases in sodium chloride and *E. coli* challenged broilers compared with all other challenged groups and the control, with no significant differences between the two groups. The LBW showed highly significant increases in the diazinon, calcium sulfate, yeast extract, lead acetate, and control groups, respectively, with no significant differences between the lead acetate and yeast extract challenged birds.

**Table-2:** Performance indices (Mean±SE) in broilers challenged with different water impurities.

Groups	LBW g	WG g	FI g	FCR	PI	WI mL	WI/FI %
G1	2224^a^±9.6	433.0^a^±7.54	443.8^e^±1.2	1.03^b^±0.02	10.3^a^±0.01	846.1^a^±12.1	1.68^abc^±0.03
G2	1923^c^±7.4	375.2^b^±5.11	498.0^b^±0.9	1.36^a^±0.01	6.9^c^±0.01	838.9^a^±12.5	1.63^bc^±0.01
G3	1880^d^±4.3	366.2^b^±2.16	496.9^b^±1.8	1.33^a^±0.01	6.7^c^±0.02	841.2^a^±11.8	1.62^c^±0.00
G4	1883^d^±5.0	367.8^b^±4.28	509.2^a^±2.0	1.40^a^±0.01	6.7^c^±0.00	869.8^a^±14.3	1.63^c^±0.02
G5	1947^b^±9.1	376.8^b^±6.74	486.6^c^±1.1	1.38^a^±0.02	7.2^c^±0.02	828.2^a^±16.3	1.64^bc^±0.01
G6	2233^a^±7.5	435.8^a^±6.23	481.5^c^±2.0	1.16^b^±0.05	9.6^b^±0.02	880.4^a^±15.2	1.73^ab^±0.00
G7	1658^e^±7.2	323.1^c^±5.47	453.7^d^±1.4	1.47^a^±0.02	6.0^d^±0.02	836.5^a^±12.4	1.74^a^±0.01
p-value	0.001	0.009	0.002	0.032	0.012	0.430	0.028

Means carrying different superscripts in the same column are significantly different at (p≤0.05) or highly significantly different at (p<0.01). Means carrying the same superscripts in the same column are non-significantly different at (p>0.05). G1=Broilers challenged with sodium chloride (700 mg/L), G2=Broilers challenged with calcium sulfate (80 mg/L), G3=Broilers challenged with lead acetate (500 mg/L), G4=Broilers challenged with yeast extract 5% (5 mg/L), G5=Broilers challenged with diazinon (2.5 mL/L), G6=Broilers challenged with *Escherichia* *coli* O157: H7 (1.6 × 10^9^ CFU/mL), G7=Control broilers. LBW=Live body weight, WG=Weight gain, FI=Feed intake, FCR=Feed conversion ratio, PI=Performance index, WI=Water intake, WI/FI=Water to feed intake ratio, SE=Standard error

Increases in WG (Table-2) were highly significant (p<0.01) in the sodium chloride and *E. coli* challenged broilers compared with all other challenged groups and the control, with no significant differences between the two groups. There were no significant differences among broilers challenged with calcium sulfate, lead acetate, yeast extract, and diazinon, but showed highly significant (p<0.01) increases compared to the control group.

Feed intakes showed highly significant (p<0.01) increases in yeast extract, calcium sulfate, lead acetate, diazinon, *E. coli*, sodium chloride, and control groups, with no significant differences between the calcium sulfate and lead acetate challenged birds or between diazinon and *E. coli* challenged broilers (Table-2). Water intake in Table-2 showed no significant differences among all challenged groups.

Feed conversions in Table-2 show highly significant (p<0.01) increases in calcium sulfate, lead acetate, yeast extract, diazinon, and control groups, with no significant differences among the five groups. *E. coli* and sodium chloride challenged broilers exhibited highly significant decreases (p<0.01) in the FCR, but with no significant differences between each other. Performance indices showed highly significant (p<0.01) increases in sodium chloride, *E. coli*, calcium sulfate, lead acetate, yeast extract, diazinon, and the control, respectively, with no significant differences between the calcium sulfate, lead acetate, yeast extract, and diazinon challenged broilers.

### Carcass, edible, and immune organ weight

Carcass weights, as shown in Table-3, revealed highly significant (p<0.01) increases in *E. coli* and sodium chloride challenged broilers compared to other challenged and control broilers. Diazinon challenged broilers exhibited highly significant (p<0.01) increases compared to calcium sulfate, lead acetate, and yeast extract challenged groups, with no significant differences among the three groups.

**Table-3:** Carcass and organ’s weights (Mean±SE) in broilers challenged with different water impurities.

Groups	CW g	Edible organ weights/g		Immune organ weights/g	
	
		Liver	Heart	Spleen	Bursa
G1	2011^a^±9.6	49.3^a^±1.68	10.2^b^±0.48	2.8^a^±0.06	1.6^a^±0.01
G2	1670^c^±4.3	44.0^bc^±0.73	12.4^a^±0.38	2.8^a^±0.01	1.6^a^±0.04
G3	1663^c^±5.4	46.4^ab^±2.11	9.4^b^±0.28	2.4^b^±0.01	1.1^cd^±0.04
G4	1691^c^±5.2	41.4^c^±0.75	9.9^b^±0.35	2.2^b^±0.09	1.4^ab^±0.06
G5	1746^b^±9.3	50.7^a^±1.69	9.3^b^±0.23	2.8^a^±0.07	1.3^bc^±0.06
G6	2029^a^±7.3	50.6^a^±1.70	9.3^b^±0.21	2.9^a^±0.05	1.3^bc^±0.06
G7	1458^d^±6.2	33.1^d^±1.87	4.7^c^±0.17	1.2^c^±0.06	1.0^d^±0.06
p-value	0.001	0.008	0.011	0.001	0.028

Means carrying different superscripts in the same column are significantly different at (p≤0.05) or highly significantly different at (p<0.01). Means carrying the same superscripts in the same column are non-significantly different at (p<0.05). G1=Broilers challenged with sodium chloride (700 mg/L), G2=Broilers challenged with calcium sulfate (80 mg/L), G3=Broilers challenged with lead acetate (500 mg/L), G4=Broilers challenged with yeast extract 5% (5 mg/L), G5=Broilers challenged with diazinon (2.5 mL/L), G6=Broilers challenged with *Escherichia coli* O157: H7 (1.6 × 10^9^ CFU/mL), G7=control broilers. CW=carcass weight, SE=Standard error

The liver weights revealed highly significant (p<0.01) increases in diazinon, *E. coli*, sodium chloride, and lead acetate challenged broilers, with no significant differences among the groups. Heart weights revealed highly significant (p<0.01) increases in calcium sulfate, sodium chloride, yeast extract, lead acetate, diazinon, *E. coli*, and control groups, with no significant differences among the sodium chloride, yeast extract, lead acetate, diazinon, and *E. coli* challenged broilers. Spleen weights revealed highly significant (p<0.01) increases in *E. coli*, sodium chloride, calcium sulfate, diazinon, lead acetate, yeast extract, and the control groups, with no significant differences among *E. coli*, sodium chloride, calcium sulfate, and diazinon challenged birds or between lead acetate and yeast extract challenged birds. Bursa weights revealed highly significant (p<0.01) increases in *E. coli*, sodium chloride, and yeast extract challenged birds, with no significant differences among the groups.

### Biochemical profile

The total protein, alanine aminotransferase, creatinine, glucose, triglycerides, and total cholesterol, as shown in Table-4, exhibited highly significant (p<0.01) reductions in all challenged groups compared to those of the control group, with highly significantly (p<0.01) lower readings recorded for *E. coli* challenged broilers.

**Table-4:** Biochemical assay (Mean±SE) in broilers challenged with different water impurities.

Groups	TP g.dL^-1^	ALT IU.L^-1^	Creat mg.dL^-1^	Gluco mg.dL^-1^	TG mg.dL^-1^	TC mg.dL^-1^
G1	6.4^f^±0.04	3.3^g^±0.04	0.35^e^±0.00	64.0^e^±0.95	86.0^g^±0.64	106.7^c^±1.32
G2	7.5^d^±0.04	4.1^e^±0.03	0.47^d^±0.01	70.6^d^±0.60	97.8^d^±0.23	108.3^c^±0.77
G3	8.1^c^±0.07	4.5^c^±0.04	0.58^c^±0.01	84.7^c^±1.20	100.6^c^±0.21	128.1^a^±0.92
G4	6.9^e^±0.08	4.2^d^±0.05	0.50^d^±0.02	83.4^c^±0.55	95.8^e^±0.27	108.3^c^±0.93
G5	9.4^b^±0.08	4.8^b^±0.03	0.72^b^±0.01	89.8^b^±0.85	102.4^b^±0.17	115.0^b^±0.81
G6	7.5^d^±0.05	3.6^f^±0.04	0.39^e^±0.02	70.6^d^±0.87	90.3^f^±0.37	81.8^d^±1.16
G7	9.7^a^±0.05	5.0^a^±0.03	0.98^a^±0.01	99.4^a^±0.93	104.7^a^±0.19	127.5^a^±1.02
p-value	0.002	0.000	0.003	0.001	0.000	0.002

Means carrying different superscripts in the same column are significantly different at (p≤0.05) or highly significantly different at (p<0.01). Means carrying the same superscripts in the same column are non-significantly different at (p<0.05). G1=Broilers challenged with sodium chloride (700 mg/L), G2=Broilers challenged with calcium sulfate (80 mg/L), G3=Broilers challenged with lead acetate (500 mg/L), G4=Broilers challenged with yeast extract 5% (5 mg/L), G5=Broilers challenged with diazinon (2.5 mL/L), G6=Broilers challenged with *Escherichia coli* O157: H7 (1.6 × 10^9^ CFU/mL), G7=control broilers. TP=Total protein, ALT=Alanine aminotransferase, Creat=Creatinine, Gluco=Glucose, TG=Triglycerides, TC=Total cholesterol, SE=Standard error

The total protein (Table-4) showed highly significant (p<0.01) reductions in sodium chloride, yeast extract, calcium sulfate, *E. coli*, lead acetate, and diazinon challenged broilers. Alanine aminotransferase (Table-4) showed highly significant (p<0.01) reductions in sodium chloride, *E. coli*, calcium sulfate, yeast extract, lead acetate, and diazinon challenged broilers. Creatinine in Table-4 showed highly significant (p<0.01) reductions in *E. coli*, sodium chloride, calcium sulfate, yeast extract, lead acetate, and diazinon challenged broilers, with no significant differences between *E. coli* and sodium chloride challenged broilers. Glucose (Table-4) showed highly significant (p<0.01) reductions in sodium chloride, *E. coli*, calcium sulfate, yeast extract, lead acetate, and diazinon challenged broilers. Triglycerides (Table-4) showed highly significant (p<0.01) reductions in sodium chloride, *E. coli*, yeast extract, calcium sulfate, lead acetate, and diazinon-challenged broilers. The total cholesterol showed highly significant (p<0.01) reductions in *E. coli*, sodium chloride, calcium sulfate, yeast extract, diazinon, and lead acetate challenged broilers, as shown in Table-4.

### Stress markers and immunoglobulin concentration

Cortisol sera levels, as shown in Table-5, revealed highly significant (p<0.01) reductions in the sodium chloride, *E. coli*, calcium sulfate, yeast extract, lead acetate, and diazinon challenged broilers compared to those of the control group, with no significant differences between the lead acetate and diazinon challenged broilers.

**Table-5:** Hormonal assay, immunological concentrations, and antioxidant activities (Mean±SE) in broilers challenged with different water impurities.

Groups	Cort mcg/dL	Immunoglobulins		Antioxidant activities		
	
		IgG mg.dL^-1^	IgM mg.dL^-1^	TAC mM.L^-1^	MDA nmol.mL^-1^	SOD U.mL^-1^
G1	8.6^f^±0.01	1857.6^bc^±2.52	551.4^a^±0.80	2.34^a^±0.00	42.1^a^±0.39	282.6^e^±1.91
G2	11.2^d^±0.01	1914.0^b^±1.17	537.6^c^±0.73	2.25^b^±0.00	38.2^b^±0.28	306.0^d^±0.50
G3	17.9^b^±0.02	1828.6^c^±1.82	520.0^e^±0.71	2.07^e^±0.00	29.8^d^±0.21	313.6^b^±0.31
G4	12.2^c^±0.01	1872.0^bc^±2.75	535.3^d^±0.47	2.19^c^±0.00	38.2^b^±0.22	309.7^c^±0.80
G5	17.4^b^±0.01	1817.6^c^±1.65	509.9^f^±1.06	2.10^d^±0.00	32.2^c^±0.68	320.1^a^±0.59
G6	9.7^e^±0.02	1970.0^a^±3.30	548.1^b^±0.60	2.34^a^±0.00	42.1^a^±0.41	282.6^e^±1.91
G7	19.5^a^±0.04	1687.2^d^±4.65	498.9^g^±1.03	0.97^f^±0.01	16.8^e^±0.34	146.5^f^±1.98
p-value	0.001	0.019	0.000	0.002	0.003	0.000

Means carrying different superscripts in the same column are significantly different at (p≤0.05) or highly significantly different at (p<0.01). Means carrying the same superscripts in the same column are non-significantly different at (p>0.05). G1=Broilers challenged with sodium chloride (700 mg/L), G2=Broilers challenged with calcium sulfate (80 mg/L), G3=Broilers challenged with lead acetate (500 mg/L), G4=Broilers challenged with yeast extract 5% (5 mg/L), G5=Broilers challenged with diazinon (2.5 mL/L), G6=Broilers challenged with *Escherichia coli* O157: H7 (1.6 × 10^9^ CFU/mL), G7=Control broilers. Cort=Cortisol hormone, IgG=Immunoglobulin G, IgM=Immunoglobulin M, TAC=Total antioxidant capacity, MDA=Malondialdehyde, SOD=Superoxide dismutase, SE=Standard error

IgG, as shown in Table-5, revealed highly significant (p<0.01) increases in all challenged groups compared to the control group, with no significant differences among the sodium chloride, calcium sulfate, and yeast extract challenged birds, and no significance among the sodium chloride, lead acetate, yeast extract, and diazinon challenged broilers. Meanwhile, IgM showed that highly significant (p<0.01) increases in sodium chloride, *E. coli*, calcium sulfate, yeast extract, lead acetate, and diazinon challenged broilers compared to that of the control group, as shown in Table-5.

The total antioxidant capacity (Table-5) showed highly significant (p<0.01) increases in sodium chloride, *E. coli*, calcium sulfate, yeast extract, diazinon, and lead acetate challenged broilers compared to that of the control group, with no significant differences between sodium chloride and *E. coli* challenged birds. Malondialdehyde showed highly significant (p<0.01) increases in sodium chloride, *E. coli*, calcium sulfate, yeast extract, diazinon, and lead acetate challenged broilers compared to that of the control group, with no significant differences between sodium chloride and *E. coli* or between calcium sulfate and yeast extract challenged birds, as shown in Table-5. Superoxide dismutase, as shown in Table-5, exhibited highly significant (p<0.01) reductions in sodium chloride, *E. coli*, calcium sulfate, yeast extract, lead acetate, and diazinon challenged broilers.

### Bacterial counts

The TBC of intestinal swabs and breast muscles in Table-6 showed highly significant (p<0.01) reductions in sodium chloride, *E. coli*, calcium sulfate, yeast extract, lead acetate, and diazinon challenged broilers. The TEC of intestinal swabs, as shown in Table-6, exhibited highly significant (p<0.01) reductions in *E. coli*, sodium chloride, yeast extract, calcium sulfate, lead acetate, and diazinon challenged broilers, while the TEC of breast muscles, as shown in Table-6, showed highly significant (p<0.01) reductions in sodium chloride, *E. coli*, yeast extract, calcium sulfate, lead acetate, and diazinon challenged broilers. The TLC, as shown in Table-6, exhibited highly significant (p<0.01) increases in all challenged broilers compared to that of the control group, with no significant differences among the *E. coli*, calcium sulfate, and lead acetate challenged broilers or among sodium chloride, lead acetate, yeast extract, and diazinon challenged broilers.

**Table-6:** Bacterial counts (Log_10_Mean±SE) in broilers challenged with different water impurities.

Groups	Intestinal swabs			Breast muscles	
	
	TLC CFU.mL^-1^	TBC CFU.mL^-1^	TEC CFU.mL^-1^	TBC CFU.mL^-1^	TEC CFU.mL^-1^
G1	4.53^b^±0.006	3.99^g^±0.001	1.99^f^±0.002	3.27^g^±0.003	1.80^g^±0.003
G2	4.55^a^±0.003	4.10^e^±0.005	2.06^d^±0.002	3.36^e^±0.010	1.88^d^±0.002
G3	4.54^ab^±0.001	4.16^c^±0.003	2.08^c^±0.001	3.44^c^±0.005	1.91^c^±0.001
G4	4.53^b^±0.001	4.11^d^±0.004	2.01^e^±0.001	3.41^d^±0.003	1.83^e^±0.002
G5	4.52^b^±0.002	4.20^b^±0.005	2.10^b^±0.001	3.49^b^±0.005	1.92^b^±0.001
G6	4.55^a^±0.001	4.00^f^±0.008	1.98^g^±0.002	3.29^f^±0.006	1.81^f^±0.002
G7	4.26^c^±0.002	4.28^a^±0.004	2.11^a^±0.003	3.55^a^±0.010	1.95^a^±0.004
p-value	0.009	0.000	0.001	0.001	0.000

Means carrying different superscripts in the same column are significantly different at (p≤0.05) or highly significantly different at (p<0.01). Means carrying the same superscripts in the same column are non-significantly different at (p>0.05). G1=Broilers challenged with sodium chloride (700 mg/L), G2=Broilers challenged with calcium sulfate (80 mg/L), G3=Broilers challenged with lead acetate (500 mg/L), G4=Broilers challenged with yeast extract 5% (5 mg/L), G5=Broilers challenged with diazinon (2.5 mL/L), G6=Broilers challenged with *Escherichia coli* O157: H7 (1.6 × 10^9^ CFU/mL), G7=control broilers. TLC=total Lactobacillus count, TBC=Total bacterial counts, TEC=Total Enterobacteriaceae count, SE=Standard error

### Histopathological Architecture

**Figure-1:**
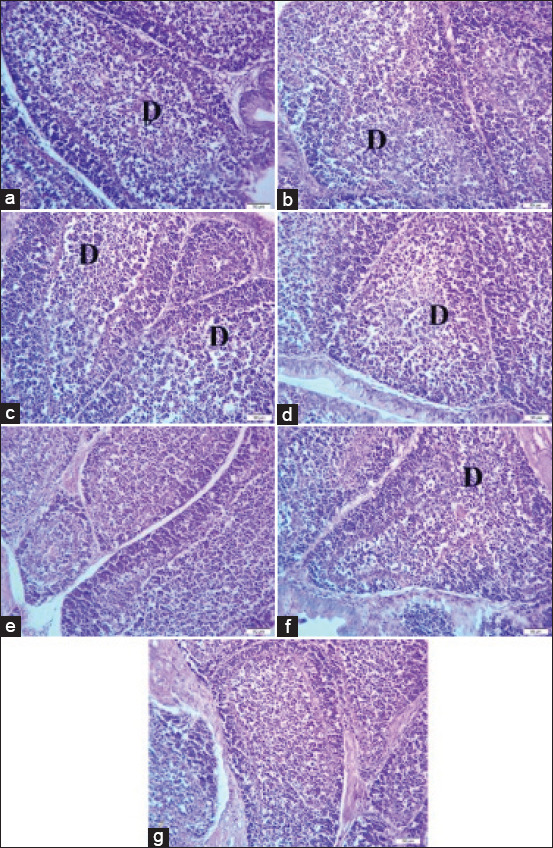
(a) Bursa of Fabricius of broiler chickens challenged with sodium chloride (700 mg/L), (b) Bursa of Fabricius of broiler chickens challenged with calcium sulfate (80 mg/L). (c) Bursa of Fabricius of broiler chickens challenged with lead acetate (500 mg/L). (d) Bursa of Fabricius of broiler chickens challenged with yeast extract 5% (5 mg/L). (e) Bursa of Fabricius of broiler chickens challenged with diazinon (2.5 mL/L). (f) Bursa of Fabricius of broiler chickens challenged with *Escherichia coli O157: H7* (1.6 × 10^9^ CFU/mL). (g) Bursa of Fabricius of control broiler chickens. Where D indicates lymphoid depletion. H&E, 40×, Bar is 50 μm.

Photomicrographs of the bursa of Fabricius in Figure-1 reveal mild-to-moderate degeneration of lymphoid follicles in broilers challenged with sodium chloride (Figure-1a), calcium sulfate (Figure-1b), lead acetate (Figure-1c), yeast extract 5% (Figure-1d), or *E. coli* (Figure-1f). Bursa of Fabricius of broiler chickens challenged with diazinon exhibited a nearly identical histological picture, as shown in Figure-1e, compared to the normal picture of the bursa of Fabricius, as shown in Figure-1g.

**Figure-2:**
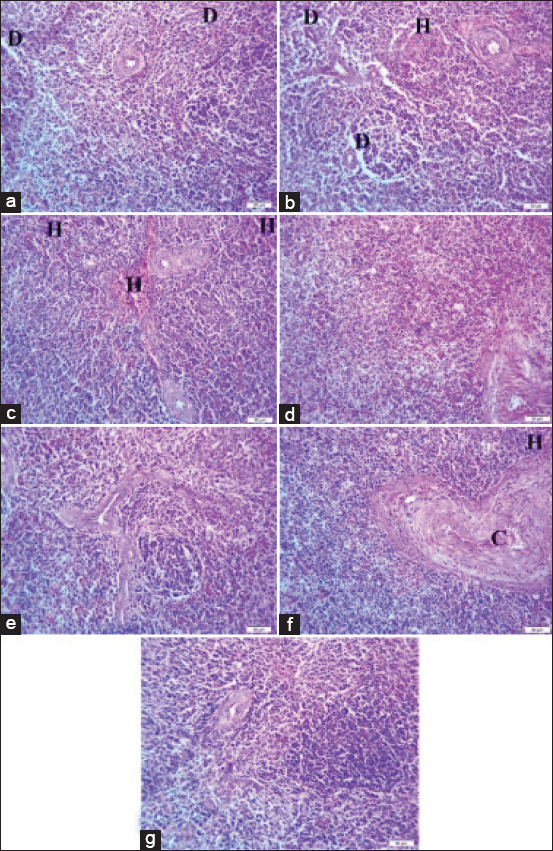
(a) The spleen of broiler chickens challenged with sodium chloride (700 mg/L), (b) spleen of broiler chickens challenged with calcium sulfate (80 mg/L). (c) The spleen of broiler chickens challenged with lead acetate (500 mg/L). (d) The spleen of broiler chickens challenged with yeast extract 5% (5 mg/L). (e) The spleen of broiler chickens challenged with diazinon (2.5 mL/L). (f) The spleen of broiler chickens challenged with *Escherichia coli O157: H7 (*1.6 × 10^9^ CFU/mL). (g) The spleen of control broiler chickens. Where D indicates lymphoid depletion, C indicates congestion of splenic sinus, and H indicates hemorrhage. H&E, 40×, Bar is 50 mm.

Spleen histopathological examination revealed mild lymphoid depletion in broilers challenged with sodium chloride (Figure-2a). The spleen of broilers challenged with calcium sulfate (Figure-2b) and lead acetate (Figure-2c) showed mild lymphoid depletion and a mild area of hemorrhage. Broilers challenged with yeast extract 5% and diazinon showed nearly normal histological pictures, as exhibited in Figures-2d and e, respectively. Challenge with *E. coli* contributed to a normal splenic histological picture with only congestion of splenic sinus and a mild area of hemorrhage, as shown in Figure-2f, compared with the normal histological appearance of the spleen in Figure-2g.

**Figure-3:**
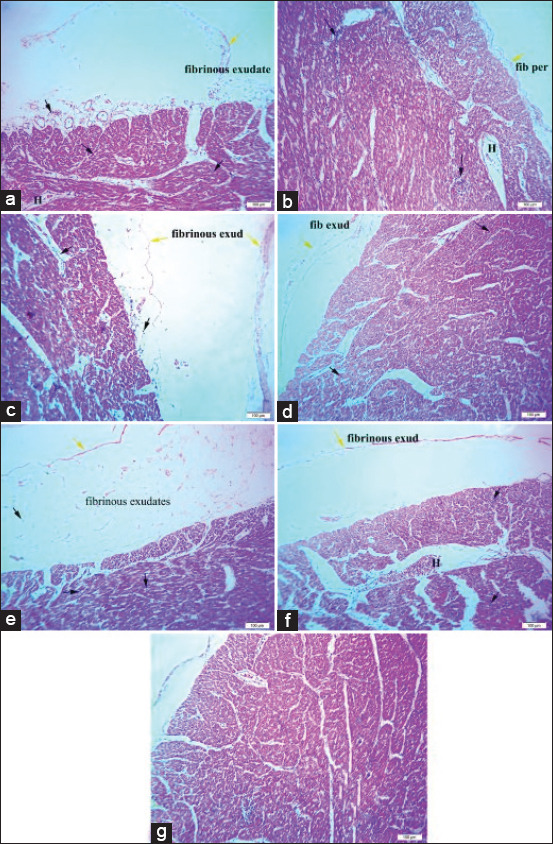
(a) The heart of broiler chickens challenged with sodium chloride (700 mg/L), (b) The heart of broiler chickens challenged with calcium sulfate (80 mg/L). (c) The heart of broiler chickens challenged with lead acetate (500 mg/L). (d) The heart of broiler chickens challenged with yeast extract 5% (5 mg/L). (e) The heart of broiler chickens challenged with diazinon (2.5 mL/L). (f) The heart of broiler chickens challenged with *Escherichia coli O157: H7* (1.6 × 10^9^ CFU/mL). (g) The heart of control broiler chickens. Where the black arrow indicates leukocytic infiltrations, the yellow arrow indicates fibrinous pericarditis, and H indicates hemorrhage. H&E, 40×, Bar is 100 μm.

Histopathological examination of the heart revealed mild fibrinous pericarditis, mild degeneration of the myocardium with mononuclear cell infiltration, and mild hemorrhage in broilers challenged with sodium chloride (Figure-3a), calcium sulfate (Figure-3b), or *E. coli* (Figure-3f). The heart of broilers challenged with lead acetate (Figure-3c) or yeast extract 5% (Figure-3d) exhibited mild fibrinous pericarditis and myocardium with mild mononuclear cell infiltrations. The heart of broilers challenged with diazinon, as shown in Figure-3e, showed moderate thickening of the pericardium with fibrinous exudate and leukocytic infiltrations, and mild myocarditis compared to the normal histological architecture, as shown in Figure-3g.

**Figure-4:**
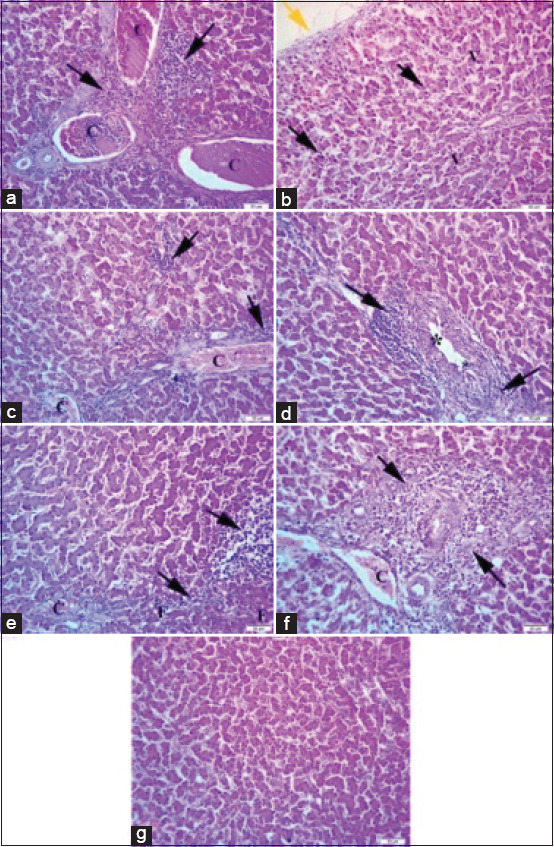
(a) The liver of broiler chickens challenged with sodium chloride (700 mg/L), (b) The liver of broiler chickens challenged with calcium sulfate (80 mg/L). (c) The liver of broiler chickens challenged with lead acetate (500 mg/L). (d) The liver of broiler chickens challenged with yeast extract 5% (5 mg/L). (e) The liver of broiler chickens challenged with diazinon (2.5 mL/L). (f) The liver of broiler chickens challenged with *Escherichia coli O157: H7 (*1.6 × 10^9^ CFU/mL). (g) The liver of control broiler chickens. Where the black arrow indicates leukocytic infiltrations, the yellow arrow indicates perihepatitis, F indicates fatty degeneration, C indicates congestion of blood vessels, and V indicates vacuolar degeneration. H&E, 40×, Bar is 50 mm.

Liver photomicrographs (Figure-4a) revealed severe congestion of portal blood vessels with perivascular mononuclear cells infiltrations within the portal area in broilers challenged with sodium chloride. The liver of broilers challenged with calcium sulfate (Figure-4b) showed mild perihepatitis, and hepatocytes showed mild vacuolation with mild leukocytic infiltrations. Hepatocytes of broilers challenged with lead acetate and yeast extract 5%, as shown in Figures-4c and d, respectively, exhibited a well-formed lobular structure and normal hepatic cord with prominent parenchymatous cells, and the portal area showed moderate leukocytic infiltrations and congestion of portal blood vessels, as well as hyperplasia of the bile duct epithelium in broilers challenged with yeast extract 5% (Figure-4d). Hepatocytes of broilers challenged with diazinon (Figure-4e) showed a moderate fatty change, vacuoles of regular boundaries, focal mononuclear cell infiltrations, and mild congestion of the central vein. The liver of broilers challenged with *E. coli* in Figure-4f revealed mild degeneration of hepatocytes, mild congestion of portal blood vessels, and severe local extensive infiltrations of mononuclear cells. Histopathological examination of liver sections from control birds, as shown in Figure-4g, revealed a well-formed lobular structure and normal hepatic lobules with prominent parenchymatous cells.

**Figure-5:**
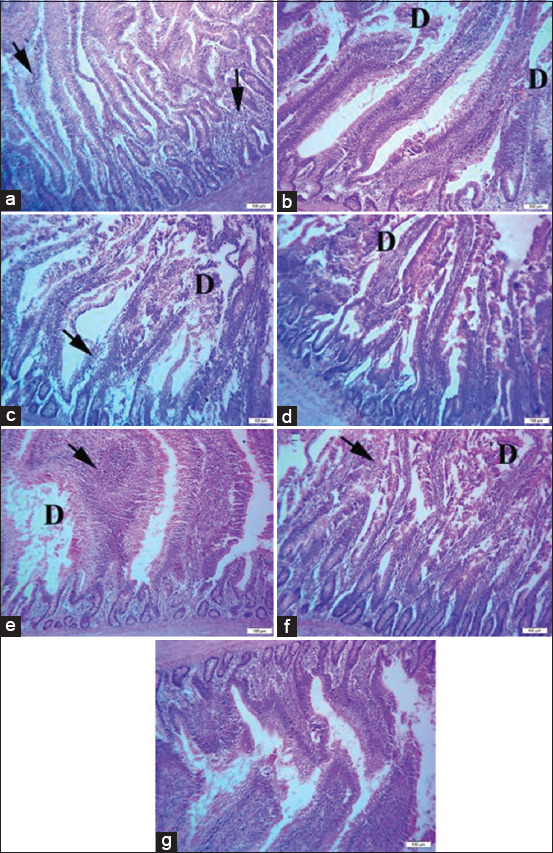
(a) The intestine of broiler chickens challenged with sodium chloride (700 mg/L), (b) The intestine of broiler chickens challenged with calcium sulfate (80 mg/L). (c) The intestine of broiler chickens challenged with lead acetate (500 mg/L). (d) The intestine of broiler chickens challenged with yeast extract 5% (5 mg/L). (e) The intestine of broiler chickens challenged with diazinon (2.5 mL/L). (f) The intestine of broiler chickens challenged with *Escherichia coli O157: H7* (1.6 × 10^9^ CFU/mL). (g) The intestine of control broiler chickens. Where the black arrow indicates leukocytic infiltrations and D indicates degeneration of lining epithelium. H&E, 40×, Bar is 100 μm.

The intestinal sections of broilers challenged with sodium chloride (Figure-5a), calcium sulfate (Figure-5b), or yeast extract 5% (Figure-5d) revealed maintained intestinal villi with an increased length of villi, mild-to-moderate degeneration of lining epithelium, and moderate leukocytic infiltrations. The intestine of broilers challenged with lead acetate (Figure-5c), diazinon (Figure-5e), or *E. coli* (Figure-5f) showed increased length and fusion of villi, with mild-to-moderate degeneration of intestinal villi and leukocytic infiltrations compared to the normal intestinal villi of the control broilers, as shown in Figure-5g.

**Figure-6:**
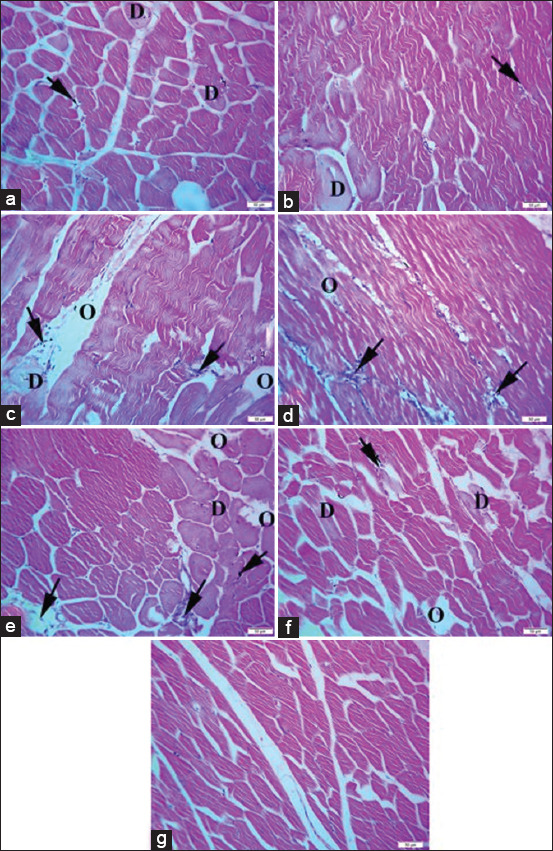
(a) The breast muscle of broiler chickens challenged with sodium chloride (700 mg/L), (b) The breast muscle of broiler chickens challenged with calcium sulfate (80 mg/L). (c) The breast muscle of broiler chickens challenged with lead acetate (500 mg/L). (d) The breast muscle of broiler chickens challenged with yeast extract 5% (5 mg/L). (e) The breast muscle of broiler chickens challenged with diazinon (2.5 mL/L). (f) The breast muscle of broiler chickens challenged with *Escherichia coli O157: H*7 (1.6 × 10^9^ CFU/mL). (g) The breast muscle of control broiler chickens. Where the black arrow indicates leukocytic infiltrations, O indicates edema, and D indicates Zenker’s degeneration. H&E, 40×, Bar is 50 μm.

Breast muscle photomicrographs revealed few muscle bundles, which indicated the loss of striations and Zenker’s necrosis, with mild leukocytic infiltration between the muscle bundles in broilers challenged with sodium chloride, as shown in Figure 6a, and calcium sulfate, as shown in Figure-6b. The intestines of broilers challenged with lead acetate (Figure-6c) or *E. coli* (Figure-6f) showed mild Zenker’s degeneration of muscle bundles, mild-to-moderate edema, and leukocytic infiltration between muscle bundles. Broilers challenged with yeast extract 5% (Figure-6d) showed mild edema and mild leukocytic infiltration between muscle bundles. Broilers challenged with diazinon (Figure-6e) showed extensive local loss of striation and Zenker’s necrosis of muscle fibers, moderate edema, and mild leukocytic infiltration between muscle bundles compared to normal muscle fibers and architecture of the control broiler group, as shown in Figure-6g.

## Discussion

Poultry requires drinking water free from pathogens, a condition that can be achieved through sterilization and transformation of water into a state known as “dead water.” Magnetization can reshape this dead water into a live state again [44]. Magnetization changes water physical characteristics with an increase of alkaline molecules (OH^-^), which contributes to an increase in water electrical conductivity, as recorded by Al-Nuemi *et al*. [45]. Esmaeilnezad *et al*. [46] and Yacout *et al*. [47] revealed that magnetization was effective compared to other methods used to improve water quality and increased the dissolving capacity for different nutritive substances, such as minerals and vitamins, which contributed to a positive influence on performance, with a significant change in the total dissolved solids, pH, total alkalinity, dissolved oxygen, organic matter, and TBC [48].

In the current study, magnetizing water using a 13,200 gausses magnetic field contributed to significant changes in water quality and, thus, significant increases in LBW, weekly WG, feed intake, PI, and water intake in broilers, regardless of the environmental water challenges introduced to the birds weekly. The enhancing influence on performance traits might be attributed to the influence of magnetic water in increasing cell permeability, which causes an expansion in the gastrointestinal tract, thereby increasing feed utilization and improving the absorption of nutrient substances, as revealed by Oyngi *et al*. [49]. In addition, the results showed highly significant improvements in the *Lactobacillus* count, reflect the improvement in gastrointestinal tract conditions. Gholizadeh *et al*. [50] recorded increases in the bodyweights of the magnetic water-treated group by approximately 200 g compared to that of the control group. They also recorded increases in the livability, production efficiency, meat/fat ratios, quality of the final product, reductions in mortalities, and feed reduction cases.

Ahmed *et al*. [51] recorded that magnetized water significantly improved the final body weight, daily WG, FCR, protein efficiency ratio, and PI by approximately 7.3%, 7.4%, 11.7%, 23.3%, and 20.6% for 1-day-old Cobb broilers. Gilani *et al*. [52] showed that water magnetized with a magnetizer of 0.65 T (6500 gausses) for 3 h lead to greater water consumption during the trial period, with significantly increased feed intake and WG in chickens during the starting phase. However, magnetic water did not influence the FCR, mortalities, European production efficiency factor, or bio-economic index. Al-Fadul [53] stated that water magnetization significantly improved body weight, body WG, and feed efficiency while reducing water consumption, especially during the late weeks, in a day-old Arbor Acres breed. Mitre [54] observed no significant improvements in the performance parameters of Cobb-500 chicks consuming magnetized water in comparison with groups consuming untreated water, and so determined that magnetizing the water was not beneficial for broilers receiving a dietary treatment that meets their nutritional needs.

Alhassani and Amin [55] recorded no significant differences in the body weight, WG, mortality rate, feed intake, FCR, performance index, and viability of Cobb-500 broiler chicks treated with magnetic water using a magnetizer of 500 gausses with 5, 10, and 15 min speeds; however, the contradiction might be attributed to the strength of the magnetic field used. El-Katcha *et al*. [56] recorded an improved feed conversion and PI in Pekin ducklings and attributed these improvements to the ability of magnetic water to increase the intestinal villi length, thus improving nutrient absorption and body weight.

Our results also revealed significant improvements in the weights of the carcass, some immune organs (spleen and bursa), and edible organs (heart and liver) in all challenged broilers treated with magnetic water. These results agree with those of Hassan *et al*. [57], who found significantly higher feed intakes and better feed conversions in Gimmizah chickens receiving magnetized water of 2000, 3000, and 4000 gausses compared to those of the control group, which is a condition attributed to higher levels of triiodothyronine (T3). They also recorded increases in egg weights, albumin percentage, albumin dry matter, yolk, and shell thickness.

The current study recorded significant reductions in the serum levels of most measured biochemical parameters, including total protein, alanine aminotransferase, creatinine, glucose, total cholesterol, and triglycerides in all challenged groups compared to those of the control group. Khalisa and Aous [58] supported our results when they reported significantly good levels of total protein, albumin, liver enzymes, and creatinine in rabbits treated with magnetic water compared to those of the non-treated group. The current results were consistent with those of Al-Hilali [59], who recorded significant reductions in glucose, total cholesterol, and triglycerides levels in Japanese quail treated with magnetic water compared to those of the control group. He attributed these reductions to the capability of magnetic water to increase protein and lipid metabolism and induce hypotriglyceridemia. Mahmoud *et al*. [60] also reported that magnetic water did not influence the serum levels of liver enzymes (alanine aminotransferase and aspartate aminotransferase). Jassim and Aqeel [61] recorded significant reductions in blood glucose, cholesterol, and triglycerides up to 115 mg/dL, 142.9 mg/dL, and 151.8 mg/dL, respectively, in 160 Ross^®^ broiler chickens treated with magnetic water.

Our results revealed significant reductions in cortisol hormone in the blood, even though in broilers challenged with water impurities in different forms, the lower levels of cortisol might explain the normal levels of glucose in all challenged birds compared to those of the control group. Magnetic water also significantly reduced antioxidants, as evidenced by the total antioxidant capacity, malondialdehyde levels, and superoxide dismutase, exhibiting minimized action of magnetic water for all stress factors (water impurities) introduced to all challenged broilers. These results were consistent with those of Saleh *et al*. [62], who recorded that magnetic water could improve antioxidant levels in type 2 diabetic laboratory animals. Attia *et al*. [63] recorded significant reductions in lipid peroxidation markers, as malondialdehyde and thiobarbituric acid-reactive substances were associated with increases in red blood cells and lymphocytes in rabbit buck treated with magnetized water exposed to a field of 4000 gausses, which improved rabbit buck fertility. Hafizi-Lotfabadi *et al*. [64] also stated that magnetic water could minimize antioxidant levels in rats supplemented with magnetic water by minimizing hepatic cellular deoxyribonucleic acid and maintaining cellular integrity.

The results revealed significant increases in the serum levels of total IgG and IgM, thus indicating an increase in the immune status of the birds and higher efficiency of the used vaccines. Our results were consistent with those of Saeed and AlShidede [65], who recorded improvements in the blood picture and increases in blood ion concentrations, which indicated higher chemical reactions, increases in blood vitamins and minerals, and significant increases in immunity. Abd El-Hamid *et al*. [66] also recorded that magnetic water could inflate the sera concentrations of IgG, IgM, and IgA in V-line male rabbits supplemented with magnetic water from 4000 gausses magnetic field. El-Sabrout and El-Hanoun [67] found that magnetic water with a high gauss, particularly over 1000 gauss, could improve bird health and immunity. El-Hanoun *et al*. [68] reported that magnetizing water with a 6000 gausses magnetic field enhanced the water quality, thus contributing to highly significant increases of the IgG, IgM, and IgA concentrations of male geese consuming the magnetized water. Glowinska *et al*. [69] confirmed that magnetized drinking water did not affect blood constituents such as potassium and chloride or the health and performance of guinea fowl.

Our results also revealed a significant reduction in the TBC and TEC of intestinal swabs and breast muscles, which indicated the high resistance of birds and neutralization of infection by most pathogenic microorganisms, like *E. coli*, and the direct *in vitro* capability of magnetic water to reduce *E. coli* O157:H7 and *S*. Typhimurium survival by 54.91% and 39.89%, respectively, after 4 h. The results were consistent with El-Katcha *et al*. [70], who recorded that magnetic water supplementation in Cobb-500 broiler chicks challenged with *Salmonella enteritidis* could overcome the negative consequences of *Salmonella* infection, improve immunity, and enhance performance.

Histopathological examination in the current study confirmed that magnetic water could minimize the influence of all induced challenges, including sodium chloride (700 mg/L), calcium sulfate (80 mg/L), lead acetate (500 mg/L), yeast extract 5% (5 mg/L), diazinon (2.5 mL/L), and *E. coli* O157:H7 (1.6 × 10^9^ CFU/mL) applied on the examined organs, including bursa of Fabricius, spleen, heart, liver, intestine, and breast muscles. These results were consistent with the results of El-Katcha *et al*. [70], who revealed that magnetic water treatment contributed to non-significant increases in the length and width of ileum villi by up to 7.5% and 10.9%, respectively, with a reduction in the crypt’s depth by up to 9.7%. They also reported that the usage of magnetic water or a combination of magnetic and acidified water could minimize the impact of *Salmonella* infection used in the study, as well as improve performance and immunity levels.

The current results also revealed that treating all challenged groups with magnetic water contributed to a pronounced increase in the length of villi, with a fusion of villi, and increased surface areas as compared to those of the control group. These data are probably the main reason for improvements in the absorption of intestinal nutrients and increased enteric functions. These results were consistent with the results reported by El-Katcha *et al*. [56], who showed increases in the length of intestinal villi, thus indicating improved nutrient absorption and increased body weights of growing Pekin ducklings compared with those of the control group. Saleh *et al*. [62] also observed significant enhancements of the pancreas in diabetic pancreatic rats treated with magnetic water, which exhibited a final size and cellular structures of islets of Langerhans that were nearly comparable to those of the control group. They also recorded lower development of vacuoles resulting from cytoplasmic autophagy, with nearly normal morphology of beta cells. Chung *et al*. [71] evaluated the influence of magnetic water with mono-calcium phosphate 0.5% and 1.0% as a feed additive on 108 1-day-old Cobb-500 broiler chicks. They recorded significant increases in the villi height and general gut histomorphology, which contributed to significant absorption and utilization of nutritive substances.

Histopathological examination also revealed the minimized influence of *E. coli* O157:H7 on the examined organs and tissues, which was attributed to the direct antimicrobial action of magnetic water on *E. coli*. These results agreed with the results recorded by Mahmoud *et al*. [72], who revealed the strong antimicrobial activities of magnetic water on gram-negative microorganisms such as *E. coli* and Gram-positive microorganisms as *Lactobacillus*. Broiler chickens in the 1^st^ and 2^nd^ groups of the current study exhibited significantly lower *E. coli* counts and higher *Lactobacillus* counts compared to broilers consuming white water (tap water). Gaafar *et al*. [73] recorded that magnetic water (50 hertz and 2 mT) substantially inhibited the growth rates of *E. coli* after 6 h and contributed to higher antibiotic sensitivity, with morphological deviations indicated by the decreased length of the bacterial cell; meanwhile, decreased cellular thickening with disappearance of most cytoplasmic elements was recorded after 16 h.

Verma [74] reported that magnetic water inhibited microbial growth, relieved pain, and swelling, overcame weakness, and enhanced overall health conditions. They attributed these actions to the influence of magnetization on metals, organic substances, nitrogen, and phosphorus, which are essential elements in bacterial cell reactions and metabolism. Alkhazan and Saddiq [75] stated that magnetization altered the physical and chemical properties of the water cellular structure of bacterial cells, thereby affecting their growth rates. Strasak *et al*. [76] reported significant decreases in the colonial growth of bacterial cells, as the time of exposure to magnetic water of high field strength increased. They also reported that magnetic fields act as bactericidal agents and cause decreased colonial growth and oxidoreductive activities of bacterial cells.

## Conclusion

Magnetic water in the laboratory trial maintained the viability of the Newcastle virus vaccine titer for 4 h compared to that of de-chlorinated water and physiological saline. Magnetic water also minimized the survival of *E. coli* O157:H7 and *S*. Typhimurium. At the field trial level, magnetic water minimized and modulated the influence of the induced challenges, thus significantly maintaining the biochemical parameters and improving the physiological body functions of the challenged birds that were represented in increased performance traits, carcass and immune organ weights, TLC, and sera immunoglobulin concentrations, as well as significantly reducing stress markers and TBC and TEC.

## Authors’ Contributions

ESS: Designed the experiment, conducted the *in vitro* trial, took part in the rearing of broilers, conducted the antioxidant and bacteriological examination, and took part in writing the manuscript. RTH: Conducted the histopathological examination and took part in writing the manuscript. RAH: Took part in the rearing of broilers, conducted the biochemical examination, and took part in writing the manuscript.
